# Observations on Surgery and Pathology

**Published:** 1832-07-01

**Authors:** 


### II. *bis.*

Observations in Surgery and Pathology; illustrated by Cases, and by the Treatment of some of the most important Surgical Affections. By *William Jones Clement*, Surgeon. Octavo, pp. 230. London, 1832.

Mr. Clement, the author of the volume before us, is a surgeon residing and practising at Shrewsbury. We believe that he has enjoyed a tolerably extensive private practice, and his object in publishing the present work is to communicate the results of his experience, to correct some errors which he imagines to be established, and to narrate some cases of surgical affections not commonly witnessed. Such objects cannot be otherwise than laudable, and we hope that others of our able and experienced provincial brethren will follow the example now set them by Mr. Fletcher, of Gloucester, and Mr. Clement, of Shrewsbury. The first portion of the volume consists of a critical inquiry into the different opinions respecting the structure of the urethra, and an attempt to refute the doctrines of Sir Everard Home; the second contains some cases of strangulated hernia; and the third is made up of several isolated cases—spina bifida, aneurysm by anastomosis, lithotomy in a female child, tumour attached to the biceps flexor cubiti, tumour attached to the lower jaw, and, finally, remarks on Sir Charles Bell's discovery of the functions of the facial nerves. Such are the many-coloured contents of the book, and it may be supposed that we do not pretend to offer an analytical review of them. In consequence of the narrowed limits of the review department, our notice here must be very brief; but we shall endeavour to supply, in the Periscope, an abstract of the most curious and interesting facts.

The first portion, that which treats of the urethra, need not detain us long. It is chiefly occupied with the refutation of Sir Everard Home's opinions on the muscularity of that canal. We believe that few surgeons now believe, or anatomists inculcate, such a doctrine, and it would be felling a man of straw to reiterate the arguments and proofs against it. The only part of the paper to which we think it necessary to direct attention, is one in which allusion is made to the nature and treatment of that difficulty in making water which not unfrequently succeeds gonorrhœa.

"The fears of patients are frequently excited, by the stream of urine appearing smaller than usual after their recovery from the acute symptoms of gonorrhœa; and they actually may experience some difficulty in voiding it; this they of course attribute to the formation of a stricture, whereas it more frequently depends upon the want of elasticity in the canal, produced by the previous inflammation; and some degree of thickening may probably exist as the consequence of it, but which will gradually subside, if the canal be not interfered with, or irritated, by the passing of instruments.

So convinced am I of the impropriety of, and the bad effects which arise from, the use of bougies, in what are supposed to be cases of recently-formed strictures, that whenever I am consulted by patients who have some difficulty in making water, and express their fear of the formation of a stricture, I invariably object to the introduction of the bougie, if, upon enquiry, I find that they have only recently recovered from gonorrhœa. My practice, in such cases, is to direct a small quantity of the Linim. Hydr. Camphor to be rubbed along the line of the urethra every night and morning; and it has always happened that those patients, who have had confidence to pursue this plan and give it a fair trial, have returned to me in the course of a short period, declaring themselves perfectly free from any obstruction, or difficulty in making water." 39.

If obliged to sound the patient, our author has generally found one tender point in the urethra, about three or four inches from the orifice. Both the bougie within, and pressure from without, produce a sensation of soreness and pain here, and Mr. Clement imagines that a slight degree of inflammatory action, lurking in the part, is the cause. In some examinations after death, which he has had an opportunity of making, he has found redness, vascularity, and a degree of firmness in the membrane of the urethra at this spot. In such cases, Mr. Clement avoids the bougie, although it is too frequently employed in them, under the supposition of the existence of a spasmodic stricture. Mr. Clement's plan of treatment is, to direct the application of leeches as near as possible to the part affected, and repeat them once a week for a month, or longer if circumstances require it, giving, at the same time, full doses of the liquor potassæ. When the sensation of soreness is removed, he advises the application of the linimentum hydrargyri camphoratum. After the diligent employment of this method for some time, he has been enabled to pass a full-sized bougie into the bladder without difficulty or the production of pain. We have drawn attention to this mode of practice, because we know, from experience, that it is successful. We have seen many instances of diminished stream of urine after gonorrhœa, which by mild and unirritating treatment has been removed, and has not subsequently terminated in stricture. We agree with Mr. Clement, that the bougie is not only not required in such cases, but must prove absolutely prejudicial in many.

We pass to the subject of hernia, or rather to the cases of this disease; for the remarks are only in the form of notes upon these, as they are detailed.

Case 1.—*Strangulated Inguinal Hernia—Operation—subsequent Protrusion and Mortification of Omentum.*

Mrs. B. æt. 49, who had long been subject to reducible inguinal hernia, but worn no truss, was seized with the symptoms of strangulation in the night of the 5th of November, 1823. The operation was not performed till the evening of the 6th, two large bleedings, with the warm bath and taxis, having been ineffectually employed in the interim. The contents of the hernial sac were found to be a considerable portion of omentum, and below this, three or four inches of small intestine, firmly strictured and exceedingly livid. The stricture was formed by the neck of the sac. The principal symptoms subsequent to the operation were, retention of urine for a day or two, and, on the 8th, some fever, with shooting pains in the loins and bowels. On the 9th she was better, and in two or three days the wound was, to all appearance, closed, and the patient in a very favourable condition. In straining to evacuate the bowels on the 13th, the wound opened, and a considerable piece of omentum was protruded. When the patient was visited it was impossible to reduce the omentum, now as large as a billiard-ball. It became strangulated, sloughed, and separated, without the occurrence of an unpleasant symptom. The wound was perfectly healed on the 2d of December.

Mr. Clement, like all judicious surgeons, is a warm advocate for an early operation in cases of strangulated hernia. The operation is rarely dangerous—delay almost always is so. He is also inimical to the use of the tobacco enema, because he has seen it prove fatal, because he has never seen it beneficial, because its good effects may be obtained by the warm bath and bleeding without the risk of its bad ones, and because it not only protracts the performance of the operation, but probably renders the patient worse calculated to bear it and less willing to submit to it. Mr. Clement objects to active purgatives before or after the operation. If aperients are necessary after it, they should be mild ones, and enemata are usually to be preferred.

Case 2.—*Strangulated direct, or Ventro-Inguinal Hernia—Operation—Epilepsy afterwards.*

Wm. Giles, æt. 29, iron-founder, robust and temperate, had been subject, for six years, to a small hernia, for which he wore no truss. In the morning of the 19th January, he felt the hernia descend lower, and was attacked with symptoms of strangulation. At 7, a.m. Mr. Clement saw him. He was suffering great pain. The hernial tumour was of the size of a common lemon, and, on examination, Mr. C. discovered that it was of the direct, or ventro-inguinal description. Mr. C. bled him to syncope, employed the taxis, produced syncope again in the warm bath, and again employed the taxis without success. Six hours had now elapsed since the commencement of symptoms of strangulation, and at 1, p.m. the operation was performed.

"After the integument and superficial fasciæ were freely divided, the spermatic cord was seen running obliquely across the lower part of the wound. I pushed it gently downwards, and directed my assistant to place his finger upon it. The layers of fasciæ covering the hernial sac were very thin, and it was difficult to distinguish the difference between the true peritoneal covering and the intestine which was seen through it, presenting a very livid colour.

The sac was cautiously pinched up, taking care to avoid including any portion of the intestine; then, holding the knife horizontally, I made a small opening into it, which was afterwards enlarged by the bistoury. It was not until the sac had been freely opened, that any serum escaped, and then only a very small quantity. About three inches of the jejunum was exposed, in colour more resembling a ripe plum than any thing else with which I can compare it. The greatest caution was required in passing a very fine director into the neck of the sac, which was very firm, and formed the stricture. Previous to its division, I pressed my little finger gently against it, and felt the pulsation of an artery, which, from its situation, must have been the epigastric. The neck of the sac was divided in a direction upwards, but inclining a little inwards towards the pubes." 77.

At 7, p.m. the bowels had not been opened, there was occasional sickness, the pulse 120, full and strong. *V.S. ad* ℥xxiv. blood not buffed. At 5 o'clock next morning the symptoms continued, with extreme tenderness of the abdomen; castor oil, which had been given, had not operated. *V.S. ad* ℥xxx. *Enema, salin. c. tart.-emet.* blood much buffed. The symptoms were the same in the evening, and next day the right testicle was swollen. The tenderness of the abdomen was gone—he was very low—the bowels had been opened by the enema, but not since. *Ol. ric.* ℥ss. The oil acted on him very violently, and in the evening his pulse was fluttering, and he seemed to be sinking. It required the utmost attention, with the regular exhibition of wine and water, brandy and water, &c. to enable him to rally. Ultimately the patient recovered completely. In the course of a month after the healing of the wound, the patient was seized with epilepsy. The fits occurred at first every two or three days; but the intervals between them gradually became longer, and under the administration of the pil. cupri ammoniat. they finally disappeared, and there has been no recurrence of them for the last two years.

It is probable, as Mr. Clement remarks, that the depletion resorted to and the debility which it produced were the causes of the epileptic fits. We have seen epilepsy follow great depletion and low living after injuries of the head, and have seen it disappear when tonics and better diet were employed. Whether the depletion was or was not too active in the present case, we will not pretend to say, but we are certain that depletion is sometimes employed too freely after the operation of hernia, as well as after other operations and accidents. Mr. Clement has not seen another instance of affection of the testicle after this operation. We have seen the testis slough after it in a patient in St. George's Hospital. We have also twice seen hernia humoralis after the operation of lithotomy.

The next case detailed by Mr. Clement, is, practically speaking, a very instructive one. The patient was 70 years of age, the hernia femoral, the bowels had not been opened for six days, and symptoms of strangulation had existed for five. On performing the operation the gut was found very livid but firm in structure, the coats indeed being greatly thickened; it was also firmly adherent to the neck of the sac. These adhesions were separated and the gut returned. After the operation the patient was much exhausted, and a cordial with mutton broth was administered. The pulse intermitted on the next day and continued to do so until the patient was out of danger. In the night of the fourth day the patient was seized with a rigor, and on the fifth day presented symptoms of acute pneumonia. The pulse was 94, but intermitting. She was bled to ten ounces, a blister applied, and salines with expectorants exhibited. The pneumonia was checked, and, although some sloughing took place about the wound, the patient was able to walk about in the course of a month after the operation.

Mr. Clement makes some remarks on the intermission of the pulse after the operation. The fact is, that this is a very frequent occurrence after severe operations or injuries in elderly persons. We have seen it after the operation for hernia several times, after the operation of lithotomy, after amputation, &c. Some patients recovered, others did not; and although, as we have already stated, we must even look for this symptom in old people almost as a matter of course, yet it is generally an unfavourable symptom. We may mention a case or two out of several to shew that it is by no means a necessarily fatal one. A woman, 70 years of age, had a large 'hydatid' breast removed by Mr. Brodie, at St. George's Hospital. After the operation suppuration occurred in the wound, low symptoms followed, and for many days the pulse intermitted. She recovered. A very old woman had the operation for hernia performed by Mr. Hawkins, in the same hospital. Intermitting pulse succeeded, but the patient recovered from this and from the effects of the operation, although she died bed-ridden some little time afterwards. An old woman fractured the neck of her thigh-bone. Intermissions of the pulse succeeded but ceased under generous diet and stimulants. She died however at the end of a month or six weeks. Elderly persons, or those whose constitutions are debilitated by debauchery are liable to present intermissions of the pulse when attacked by erysipelas, or diffuse cellular inflammation. The symptom is then all but fatal.

Case 4.—*Strangulated Umbilical Hernia—Operation—Death.* The patient, a very corpulent woman, aged 70, had been subject to umbilical hernia for many years but never worn a truss. The hernia became larger whilst she was endeavouring to evacuate her bowels, and soon afterwards the usual symptoms were set up. The taxis was thrice employed, she was bled freely, took purgative medicines, had two enemas, and was again bled previous to the operation. When this was performed the tumour was red and inflamed from the handling at the superior part. Mr. Clement made an incision three inches long at the side of the tumour, exposed the sac which was thin and transparent, and found that it contained intestine only. The stricture was at the umbilical opening, almost cartilaginous, and very narrow. It was divided in a direction towards the linea semilunaris. The intestine was of bright red colour ; it was gradually returned. There was excessive tenderness over the belly in the evening. She was bled to 15 ozs. and had calomel, &c. On the next day she was bled again to 14 ozs. On the following morning the pulse was fluttering, and on the next morning she died. We find no notes of a cadaveric examination, indeed our author seems to have had few opportunities of obtaining knowledge in this way, if we may judge by the fatal cases already before us. It would be a trite truism to make any observation on the necessity and value of such an addition to a fatal case.

It is true that the operation for umbilical hernia in aged women succeeds but seldom. But we think it also true, or at all events probable, that the active depletion and other treatment adopted prior to the operation were not calculated to increase the chances of success in the present case. Mr. Clement has always found umbilical herniæ to consist of portions of the colon with omentum, and never saw small intestine in the sac. In a fatal case of umbilical hernia which we lately witnessed, small intestine was contained in the sac, and we should not think this circumstance usually so rare as it has proved in Mr. Clement's practice.

"Two reasons induced me to make the first incision on the side of the tumour, and not along its upper or most prominent surface. The principal one was the inequality or irregularity of the peritoneal sac which is usually met with at the front of the rupture: indeed it generally happens that the several parts are so much consolidated that the true sac cannot be easily distinguished, although I do not agree with some writers who suppose that it is entirely wanting, or does not extend upwards so far as to form an entire covering to the contents of the tumour. The second reason was, the red and inflamed condition of the integument covering the front of the rupture, produced by the repeated and long-continued use of the taxis. If an incision were made in that part, I thought the wound would not heal by the first intention, but in all probability would be followed by sloughing: a circumstance much to be dreaded, and more particularly in this species of hernia.

I once saw a very celebrated surgeon operate in a case of large umbilical hernia:—he made an incision along the whole length of the tumour, and afterwards opened the sac to its fullest extent. An immense quantity of omentum and intestine was immediately exposed; the convolutions of the latter were exceedingly troublesome; and I well remember that two pairs of hands were required to confine them before the operator could venture to divide the stricture, and when this was accomplished, a considerable length of time elapsed before every portion of the bowel could be replaced within the abdomen." 106.

There is one danger in making the division of the stricture laterally, and that is the liability to wound the internal branch of the epigastric artery. Perhaps this is in general of little moment. We fancy that the notion of a peritoneal sac being absent in umbilical hernia is unfounded.

The next case is also instructive. The patient, Mrs. M., aged 77, very thin but active, had symptoms of strangulation in a previously reducible femoral hernia for seven days before Mr. C. saw her. In performing the operation he met with considerable difficulty in consequence of the firm adhesions of the sac and its contents. Feeling omentum above he scratched through the sac into the omentum, and pushed a bistoury up to the stricture through its substance but close to the sac. The adhesions of the omentum to the lower part and sides of the sac were necessarily torn through before the gut could be exposed. It was small, livid, but firm. It adhered to the bottom of the sac; the adhesions were divided and the intestine returned. After the operation the patient was so exhausted as to require stimulants and support. She rallied, but again sank in a day or two afterwards into an alarming state of depression, from the effects of a dose of castor oil. From this she was happily recovered, and was perfectly well in less than a month after the operation.

Here this article must terminate, not for want of other and equally useful cases, but for want of space on our parts for their insertion. Those which we shall find most adapted for our purpose will be noticed in the Periscope department. The nature of the work will be apparent to our readers; it is a plain unvarnished record of the experience of an active and practical provincial surgeon. Would that his brethren and coadjutors in all parts of the kingdom would follow his example, and rescue their names from something like the reproach of inactivity, we mean on the score of communicating their knowledge to the world, which now attaches to them. These are times for all men to bestir themselves, and the period is approaching when the field for professional exertion and professional honours must be laid more open than it is at present. Let the surgeons of the empire prove that they are worthy of attention, and if prepared to demand, they will obtain it.

